# Identification of candidate protective variants for common diseases and evaluation of their protective potential

**DOI:** 10.1186/s12864-017-3964-3

**Published:** 2017-08-03

**Authors:** Joe M. Butler, Neil Hall, Niro Narendran, Yit C. Yang, Luminita Paraoan

**Affiliations:** 1 Department of Eye and Vision Science, Institute of Ageing and Chronic Disease, University of Liverpool, 6 West Derby Street, Liverpool, L7 8TX UK; 2 The Earlham Institute, Norwich Research Park, Norwich, NR4 7UH UK; 3 Department of Ophthalmology, The Royal Wolverhampton NHS Trust, New Cross Hospital, Wolverhampton, WV10 0QP UK

**Keywords:** Candidate gene, Protective variants, Common variants, Missense mutation, Gene selection, Age-related macular degeneration, Non-synonymous SNP, Gain of function, Ancestral allele, Type 2 diabetes, Inflammatory bowel disease, Multiple sclerosis and rheumatoid arthritis

## Abstract

**Background:**

Human polymorphisms with derived alleles that are protective against disease may provide powerful translational opportunities. Here we report a method to identify such candidate polymorphisms and apply it to common non-synonymous SNPs (nsSNPs) associated with common diseases. Our study also sought to establish which of the identified protective nsSNPs show evidence of positive selection, taking this as indirect evidence that the protective variant has a beneficial effect on phenotype. Further, we performed an analysis to quantify the predicted effect of each protective variant on protein function/structure.

**Results:**

An initial analysis of eight SNPs previously identified as associated with age-related macular degeneration (AMD), revealed that two of them have a derived allele that is protective against developing the disease. One is in the complement component 2 gene (*C2*; E318D) and the other is in the complement factor B gene (*CFB*; R32Q). Then, combining genomewide ancestral allele information with known common disease-associated nsSNPs from the GWAS catalog, we found 32 additional SNPs which have a derived allele that is disease protective. Out of the total 34 identified candidate protective variants (CPVs), we found that 30 show stronger evidence of positive selection than the protective variant in lipoprotein lipase (*LPL*; S447X), which has already been translated into gene therapy. Furthermore, 11 of these CPVs have a higher probability of affecting protein structure than the lipoprotein lipase protective variant (*LPL*; S447X).

**Conclusions:**

We identify 34 CPVs from the human genome. Diseases they confer protection against include, but are not limited to, type 2 diabetes, inflammatory bowel disease, age-related macular degeneration, multiple sclerosis and rheumatoid arthritis. We propose that those 30 CPVs with evidence of stronger positive selection than the *LPL* protective variant, may be considered as priority candidates for therapeutic approaches. The next step towards translation will require testing the hypotheses generated by our analyses, specifically whether the CPV arose from a gain-of-function or a loss-of-function mutation.

**Electronic supplementary material:**

The online version of this article (doi:10.1186/s12864-017-3964-3) contains supplementary material, which is available to authorized users.

## Background

Mutations associated with diseases are usually considered to be detrimental to health, increasing the risk of disease. However there are a growing number of reported missense rare mutations shown to be protective, lowering the risk of certain diseases and conditions [1, 2]. These are beginning to present valuable therapeutic opportunities [3]. If such a mutation gives rise to a selective advantage, and given enough time, we may expect it to rise in allele frequency to become a common variant in the population. Indeed a well documented example of such a common variant exists, the S447X variant of the lipoprotein lipase (LPL). One phenotypic benefit of this derived allele is its significantly lower risk of cardiovascular diseases and hypertension [4]. Furthermore recently this variant has been utilized therapeutically for the rare condition LPL-deficiency; using a gene therapy approach the derived variant was successfully administered first in mice [5, 6] and now in humans as alipogene tiparvovec [7].

In the light of this development, we sought to identify other common protective variants (CPVs) from non-synonymous (nsSNPs) that have the potential to lead to a translational opportunity. The number of common variants associated with disease continues to grow owing to the endeavour of genome wide association studies (GWAS). The first ever GWAS was carried out for age-related macular degeneration (AMD) [8] and more recently a collaborative GWAS has identified a total of 19 AMD susceptibility loci [9]. These findings led us to begin our search for CPVs in the context of AMD. To ascertain whether a nsSNP is protective (i.e. the respective derived allele is protective) we need to deduce which allele is derived and which is ancestral. For SNPs with a very low minor allele frequency (< 1%), in a majority of the cases it can be quite safely assumed that the rare allele is the derived allele. However for common variants the assumption that the rare allele is the derived allele can be erroneous. Indeed if a derived allele provided a protective function and gives individuals’ a selective advantage one might expect positive selection to sweep it to become the most common allele in the population. Thus for all common variants we must deduce which allele is derived and which is ancestral using the genome alignments with primate species.

The identification of such protective derived variants is of importance to drug development as they represent naturally occurring therapeutic opportunities. There is a high failure rate in pharmaceutical research and development, with less than 5% of molecules entering Phase I clinical trials actually being approved as safe and effective [10], and around a quarter of those reaching Phase II trials failing due to toxicity [10]. The fact that a protective variant has existed in a part of the human population without adverse effects gives confidence that a therapeutic approach targeting them is less likely to fail in clinical trials.

An understanding of the protein function of a therapeutic candidate is an important factor in helping prioritize and direct further research and ultimately in selecting the most promising targets for a drug development programme [10]. Even before the function and mechanism of action of a protein are characterised through carefully constructed in vitro and/or in vivo molecular biology studies, it is possible to assemble some knowledge of a variant’s function using bioinformatics approaches. Specifically, if a protective variant’s function gives a selective advantage compared to the ancestral variant then we can use approaches to detect evidence of positive natural selection [11]. Accordingly, we undertook an analysis to establish which of the identified nsSNPs show evidence of positive selection, using this as indirect evidence of the protective variant having a beneficial effect on phenotype through a change in protein function.

A change in protein function can be construed to have arisen from either a gain-of-function or a loss-of-function mutation, thus leading to different translational opportunities. A protective gain-of-function mutation may be harnessed through utilizing the derived allele in a gene-therapy approach, such as in the S447X example described above. A protective loss-of-function mutation may be harnessed by therapies that inhibit the function of the ancestral gene [12]. We therefore performed a further bioinformatics analysis to assess whether the identified CPVs were derived through a loss or a gain-of-function mutation.

## Methods

### Principal logic

Our work is based on the reasoning that every biallelic SNP is composed of an ancestral allele and a derived allele. If a SNP is associated with a disease then there are two possible situations: (1) either its derived allele is associated with increased risk (being more frequent in cases than controls), or (2) its derived allele is associated with decreased risk (being more frequent in controls than cases). In the latter case, if the SNP is functionally linked to the disease (and is not just a proxy) then it may be termed a protective variant. In this study we sought to identify such protective alleles; to increase the likelihood of it being functional we limited our search to non-synonymous SNPs. We do not confirm the functional impact of these variants, and hence we cannot conclude that they are truly protective. Our study therefore identifies SNPs that they are “potentially” protective and thus we term them as *candidate* protective variants (CPVs).

The resulting list of CPVs is then subjected to various bioinformatic analyses to initiate the collection of evidence supporting their protective function and thus paving the way for the experimental characterization necessary to confirm whether they are truly protective.

### Ascertaining the ancestral and derived allele of SNPs

This ancestral allele information was inferred from whole genome alignments of 6 primate species (*H. sapiens*, *P. troglodytes*, *G. gorilla*, *P. pygmaeus*, *M. mulatta* and *C. jacchus*) released in Ensembl Compara 59 which implemented the Enredo-Pecan-Ortheus (EPO) alignment method [13]. We accessed this information from phase 1 of the 1000 Genomes project [14] by applying the *tabix* command from the SAM tools package [15] to the relevant chromosome variant call format (VCF) files.

### Identifying CPVs from previously reported AMD genetic associations

To identify CPVs from nsSNPs for AMD we began with the 19 signals reported in the most recent comprehensive meta-analysis [9]. For each signal we used the UCSC genome browser to search nearby genes for nsSNPs associated with AMD [using genome assembly GRCh37/hg19] [16]. Furthermore we only included those nsSNPs which have been validated by meta-analysis.

### Identifying CPVs from the GWAS catalog

To identify CPVs from the raw GWAS catalog we applied seven stages of filtering. Stage 1 involved selecting those associations labelled as missense. Stage 2 involved removing all associations in which the reported risk allele was missing (denoted by a “?” in the catalog). Stage 3 kept only those associations that have an ancestral allele with a high-confidence call. Stage 4 kept only those in which this ancestral allele matches the risk allele i.e. those which have a derived allele that is protective. Stage 5 kept only those associations in which an odds ratio was reported as opposed to a beta regression value (filtering out studies related to continuous traits such as height and heart rate, rather than binomial disease status). For stage 6 we manually scrutinized those studies in which associated genes were oriented on the negative strand, for such genes some studies report the risk allele as the allele on the positive strand whereas others report the coding allele on the negative strand. We removed those associations if it was not clear from the manuscript whether the risk allele referred to the positive or negative strand (note that the reported ancestral allele always refers to the allele on the positive strand). The seventh and final stage involved removing any duplicated associations, i.e. those reported in the catalog by more than one GWAS. The respective numbers of associations remaining after each of the above filter stages are presented in Additional file 1: Table S1.

### Measuring selection by calculating integrated haplotype score (iHS)

For each CPV we estimated its integrated haplotype score (iHS) using the *rehh* package [17]. This was standardized using the iHS distribution of randomly selected SNPs whose derived allele frequency matches that (within 2.5%) of the nsSNP. A negative iHS score indicates that the derived allele has undergone recent positive selection [11] and thus supports the derived allele having a function beneficial for an individual’s fitness. For this calculation we considered only common SNPs (maf > 0.01) from the chromosomal region that flank either side of the nsSNP by 100 kb. All SNP data was taken from the 1000 Genomes Project, and by combining all racial groups as a representative sample (*n* = 1092) of the human population.

We sought to increase the confidence of the iHS score by, instead of taking a single iHS value for the single nsSNP, calculating a mean iHS from a set of SNPs that will also be subject to positive selection given that the nsSNP is. This set of SNPs were defined as follows: a SNP was included in the set if satisfied all three following conditions: (i) it was in high LD with the nsSNP (pairwise D’ > 0.9) [18]; (ii) it had a derived allele that is in-phase with the derived nsSNP allele and (iii) was evolutionary younger than the nsSNP (i.e. had a derived allele frequency less than the derived nsSNP allele frequency).

A graphical way of representing these three conditions was to employ a gene or phylogenetic tree. The set defined above is equivalent to considering all those SNPs that lie on the same branch and on all branches below the nsSNP. For this purpose we implemented the package GENETREE version 9.0 [19]. Note that this program only operates under perfect phylogeny (no recombination) meaning all pairwise D’ values need to equal 1. Thus we necessarily modified our data (where all D’ > 0.9) by pruning those rare haplotypes that have occurred from recombination.

### Prediction of functional effect of variants

For 33 CPVs, we used PolyPhen-2 [20] to estimate the probability of the mutation being damaging. (One of the initial 34 CPVs (rs20541 in *IL13*) was not present in the PolyPhen-2 database and therefore is not included in this analysis.) For comparison we also included the probability for the *LPL* S447X variant. The PolyPhen-2 database (polyphen-2.2.2-whess-2011_12.sqlite) was downloaded using sqlite3. A mean probability was calculated if a mutation had multiple probabilities due to it having different effects on multiple transcripts (splice variants) of the same gene. It is important to note here that the term “damaging” used by PolyPhen-2 is meant to reflect that the mutation affects protein structure and therefore may affect function either in a gain- or loss-of-function manner. In addition the CPVs were evaluated using the Combined Annotation-Dependent Depletion (CADD) method which objectively integrates many diverse annotations to quantify the pathogenicity of human variants [21].

## Results

### Identification of two independent CPVs for AMD

We brought together eight AMD-nsSNP associations from six chromosomal loci, each of which had been validated by meta-analysis (Table 1). By ascertaining the ancestral allele for each of these eight nsSNPs we identified that two have a derived allele that is protective; inheriting either one of these alleles reduces the risk of developing AMD. One is the rs9332739 SNP in the complement component 2 gene, *C2*; the derived allele of which changes its 318th amino acid from glutamic acid to aspartic acid (E318D). The other is the rs641153 SNP in the complement factor B gene, *CFB*; the derived, protective allele changes its 32nd amino acid from arginine to glutamine (R32Q).

**Table 1:** Non-synonymous SNPs associated with AMD validated by meta-analysis

Position	Locus	nsSNP		Derived allele	Meta-analysis validation
chr1: 196,659,237	*CFH*	rs1061170:T > C	Y402H	risk	[29]
chr1: 196,642,233		rs800292:A > G	I62V	risk	[30]
chr6: 31,903,804	*C2*	rs9332739:G > C	E318D	protective	[22]
chr6: 31,914,180	*CFB*	rs641153:C > T	R32Q	protective	[22]
chr10: 124,214,448	*ARMS2*	rs10490924:G > T	A69S	risk	[31]
chr19: 6,718,387	*C3*	rs2230199:C > G	R102G	risk	[32]
chr19: 45,411,941	*APOE*	rs429358:T > C	C112R	risk	[33]
chr19: 45,412,079		rs7412:C > T	R158C	risk	[33]

It is interesting that these two nsSNP are in close physical proximity (≈10 kb apart) but are independent from one another, having low pairwise linkage disequilibrium between them (R^2^ = 0.002). It is not yet known what the level of epistasis between these two variants is upon AMD risk. Assuming no epistasis such that there is no departure from independence of effect sizes, an individual homozygous with both derived alleles (318D/318D and 32Q/32Q) is approximately 10 times less likely to develop AMD than an individual homozygous with both ancestral alleles (318E/318E and 32R/32R). This calculation is based upon the homozygote ORs reported in the relevant meta-analysis [22].

Although these SNPs are not in LD with each other, it is important to note that both these SNPs are in high LD with other nsSNPs in the region. Indeed E318D in *C2* is in almost complete LD with another nsSNP in the locus, the L9H variant (rs4151667) in *CFB* (R^2^ = 0.95). This is one reason why the AMD association information alone is not sufficient to conclude with certainty that these missense changes are functionally connected to AMD pathogenesis. Further evidence is required to conclude this.

### AMD CPVs show stronger evidence of recent positive selection than S447X in *LPL*

The fact that these AMD associated variants simply exist in the human population is testament to them not having fatal consequences or adverse effects. Thus a “natural experiment” has already been performed on these variants to test for adverse effects with zero incidences reported. This increases the appeal of them as candidates for therapeutic research and applicability.

To commence the collection of evidence supporting a functional link between these two nsSNPs and AMD pathogenesis we implemented a bioinformatics approach. Specifically we assessed the extent of recent positive selection around these nsSNPs using the integrated haplotype score (iHS) [11]. If an nsSNP has a derived allele that is protective against disease then a positive selection signal is expected given an increased reproductive fitness of those individuals inheriting such an allele. We used this method to compare the evidence for positive selection of the AMD variants against that of the previously translated *LPL* S447X variant.

**Fig. 1:**
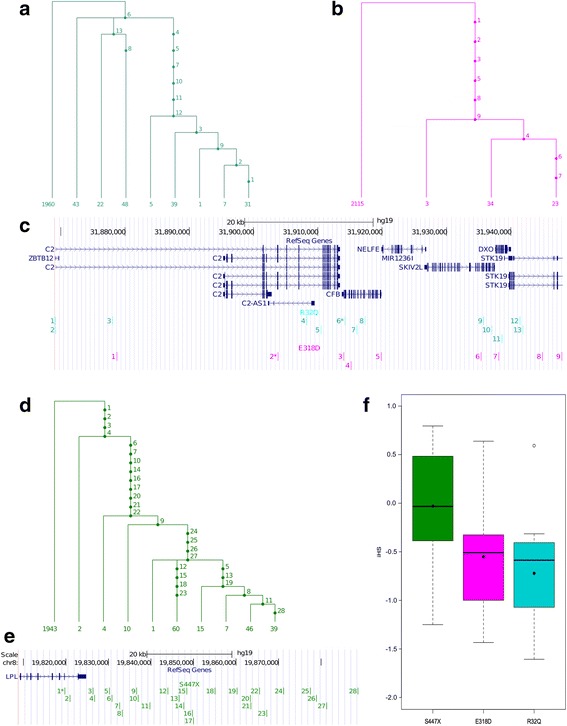
Integrated haplotype score analysis of the SNP sets corresponding to two AMD candidate protective nsSNPs in *C2* and *CFB* genes and one nsSNP protective against hypertension in *LPL*. High LD SNP sets, represented as genetic trees, for (**a**) *CFB* R32Q, (**b**) *C2* E318D and (**d**) *LPL* S447X, where SNPs are numbered on the branches. The *numbers at the bottom* of each branch represent the number of haplotypes it is comprised of. SNPs on genetic trees are aligned against UCSC genome browser (**c**) and (**e**), displaying RefSeq genes in the respective regions (nsSNPs are designated with an *asterisk* *). The integrated haplotype score (iHS) for each of these SNPs is calculated, represented by the distributions shown in (**f**) for each nsSNP. Mean iHS is represented by a *black circle* (●), median by a *thick black line* and the *box* represents the central 50% of the data. An outlier is represented by *white circle* (∘)

The variant R32Q in *CFB* presented a mean iHS of −0.72 calculated from a SNP set comprising 13 SNPs (Fig. 1a and f). For comparison, the mean iHS pertaining to the S447X in *LPL* was −0.03, calculated from a set of 28 SNPs (Fig. 1d and f). The more negative mean iHS of R32Q in *CFB* suggested it has undergone more positive selection than S447X in *LPL.* Considering that S447X has a validated beneficial functional consequence we provisionally concluded that the *CFB* R32Q variant must also have a beneficial functional consequence. Inheriting the Q allele appears to have provided a selective advantage and increases an individual’s reproductive fitness. Similarly we found the E318D variant in *C2* also gives rise to a more negative mean iHS than S447X in *LPL*. The SNP set consisting of 9 SNPs (Fig. 1b) gives a mean iHS of −0.55 (Fig. 1f). This provided support that this locus has undergone more recent positive selection than *LPL* S447X, implicating a potentially advantageous function with respect to an individual’s fitness.

We repeated this analysis using only those individuals with European ancestry from the 1000 genomes project (*n* = 379). As with the results above using all individuals, both the R32Q and E318D variants gave rise to a more negative mean iHS than S447X. Here however the E318D in *C2* presented the highest measure of selection (mean iHS = −0.74), followed by R32Q in *CFB* (mean iHS = −0.33).

### Identification of CPVs for other common diseases

**Fig. 2:**
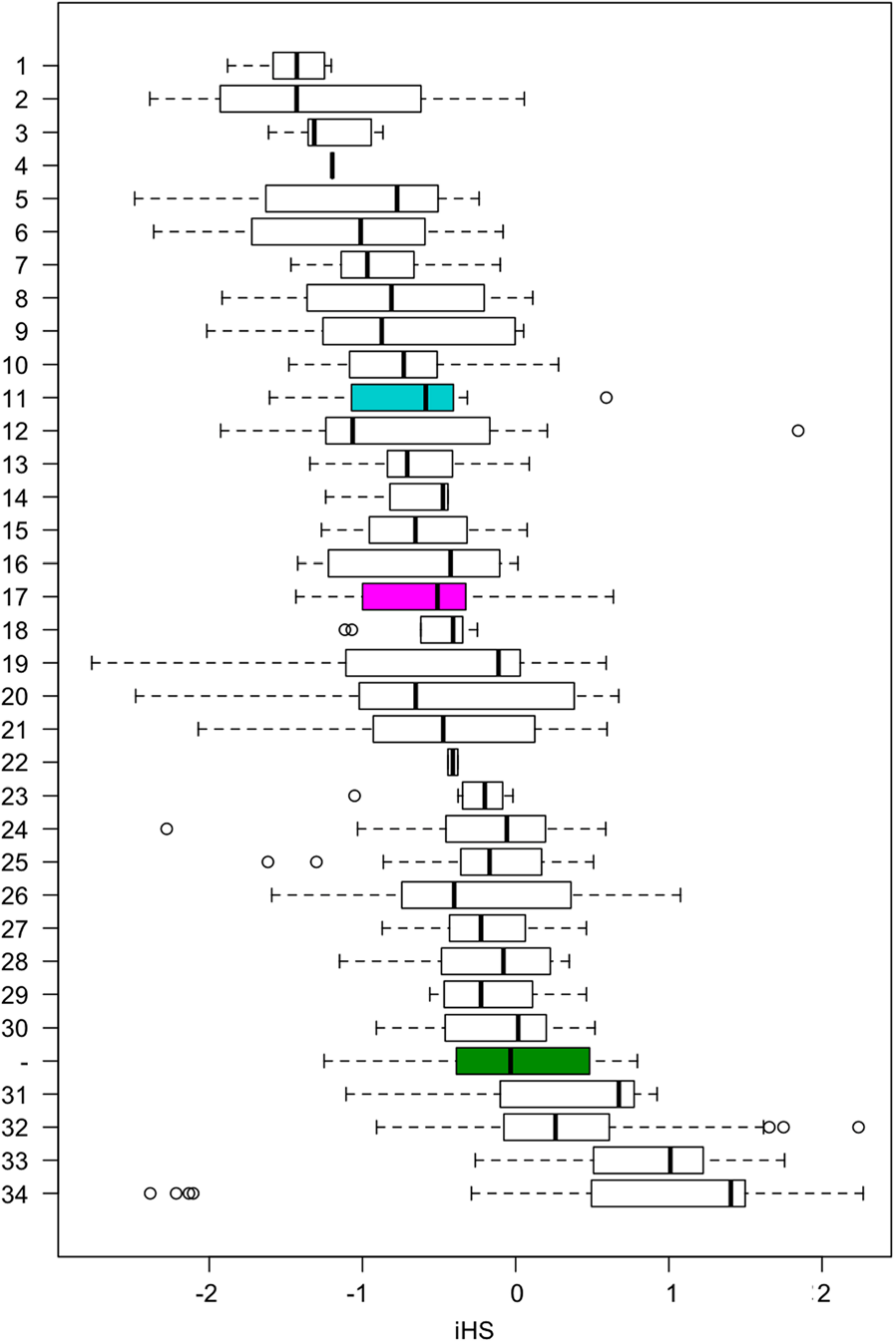
Integrated haplotype score distributions of the SNP sets corresponding to the 34 candidate protective nsSNPs. High LD SNP sets are computed for each candidate protective nsSNP, from which a distribution of the integrated haplotype scores is calculated and presented as boxplots. The distribution for *LPL* S447X is shown in *green* and those for the two AMD nsSNPs in *magenta* and *cyan*. The number identifying the nsSNP on the y-axis corresponds to the full description in Table 2

Next we applied the same reasoning to search the entire GWAS catalog for CPVs and identified a further 32 such variants, in addition to the two associated with AMD (Table 2 and Fig. 2). Out of the total 34 CPVs, 30 gave rise to a more negative mean iHS than *LPL* S447X. Four of the nsSNPs had a higher mean iHS than *LPL* S447X, and also had a positive iHS value suggesting that their derived alleles have been subject to negative selection. Taken together the number of CPVs with a mean iHS less than zero (k = 30) is significantly greater than expected by chance (*P* = 2.7 × 10^−6^; Binomial (34, 0.5) suggesting that overall, the derived allele of a CPV is more likely to have undergone positive selection than negative selection.

**Table 2:** Candidate protective nsSNPs

	Disease	Gene	rsID	Chr	Position	Anc	Der	OR^b^	SNPs in high LD set	Mean iHS	GWAS
1	Multiple sclerosis	IL7R	rs6897932	5	35,874,575	C	T	1.12	6	−1.462	[34]
2	Glaucoma (primary open-angle)	COL11A1	rs3753841	1	103,379,918	G	A	1.20	22	−1.270	[35]
3	Type 2 diabetes	SLC30A8	rs13266634	8	118,184,783	C	T	1.16	5	−1.218	[36]^a^
4	Psoriasis	TYK2	rs12720356	19	10,469,975	A	C	1.40	1	−1.197	[37]
5	Acute lymphoblastic leukemia (childhood)	KCNE4	rs12621643	2	223,917,983	T	G	1.48	3	−1.167	[38]
6	Ulcerative colitis	IL17REL	rs5771069	22	50,435,480	G	A	1.11	10	−1.121	[39]
7	Esophageal cancer and gastric cancer	PLCE1	rs3765524	10	96,058,298	T	C	1.35	23	−0.897	[40]
8	IgA nephropathy	TNFSF13	rs3803800	17	7,462,969	A	G	1.21	23	−0.819	[41]
9	Systemic lupus erythematosus	WDFY4	rs7097397	10	50,025,396	G	A	1.30	18	−0.767	[42]
10	Esophageal cancer	PLCE1	rs2274223	10	96,066,341	G	A	1.34	38	−0.740	[43]
11	AMD	CFB	rs641153	6	31,914,180	G	A	2.44	13	−0.723	-
12	Inflammatory bowel disease	CD6	rs11230563	11	60,776,209	C	T	1.09	21	−0.705	[44]
13	Behcet’s disease	KLRC4	rs2617170	12	10,560,957	T	C	1.28	46	−0.657	[45]
14	Coronary heart disease	ZC3HC1	rs11556924	7	129,663,496	C	T	1.09	6	−0.649	[46]
15	Type 1 diabetes	TYK2	rs2304256	19	10,475,652	C	A	1.16	7	−0.627	[47]
16	Obesity	GIPR	rs1800437	19	46,181,392	G	C	1.1	10	−0.606	[48]
17	AMD	C2	rs9332739	6	31,903,804	G	C	1.82	9	−0.551	-
18	Type 2 diabetes	PPARG	rs1801282	3	12,393,125	C	G	1.14	11	−0.537	[49]
19	Inflammatory bowel disease	TUBD1	rs1292053	17	57,963,537	G	A	1.08	41	−0.498	[44]
20	Migraine	MMP17	rs6598163	12	132,325,239	G	A	1.15	22	−0.493	[50]
21	Glaucoma (exfoliation)	LOXL1	rs3825942	15	74,219,582	G	A	20.1	26	−0.453	[51]
22	Inflammatory bowel disease	IL23R	rs11209026	1	67,705,958	G	A	2.01	2	−0.410	[52]
23	Multiple sclerosis	MPV17L2	rs874628	19	18,304,700	A	G	1.11	8	−0.292	[53]
24	Rheumatoid arthritis	RTKN2	rs3125734	10	63,958,112	T	C	1.20	12	−0.263	[54]
25	Interstitial lung disease	LRRC34	rs6793295	3	169,518,455	C	T	1.30	19	−0.228	[55]
26	Obesity	SH2B1	rs7498665	16	28,883,241	G	A	1.07	27	−0.210	[48]
27	Breast cancer	ANKLE1	rs8100241	19	17,392,894	G	A	1.14	11	−0.185	[56]
28	Systemic lupus erythematosus	BANK1	rs10516487	4	102,751,076	G	A	1.38	8	−0.184	[57]
29	Ovarian cancer	ANKLE1	rs2363956	19	17,394,124	T	G	1.10	13	−0.153	[58]
30	Rheumatoid arthritis	NFKBIE	rs2233434	6	44,232,920	G	A	1.20	32	−0.114	[59]
**-**	Hypertension	LPL	rs328	8	19,819,724	C	G	-	28	−0.031	**-**
31	Type 2 diabetes	WFS1	rs1801214	4	6,303,022	T	C	1.13	8	0.314	[60]
32	Type 2 diabetes	THADA	rs7578597	2	43,732,823	T	C	1.15	49	0.379	[61]
33	Psoriasis	IL13	rs20541	5	131,995,964	G	A	1.27	8	0.872	[62]
34	Prostate cancer	MLPH	rs2292884	2	238,443,226	G	A	1.14	40	0.883	[63]

^a^denotes the nsSNP has been detected by more than one GWAS in the catalog, we only report the first chronologically

^b^OR for ancestral allele

The nsSNP that had the most negative mean iHS was found in the interleukin 7 receptor (*IL7R*) gene, for which the ancestral allele codes for threonine and the derived allele codes for isoleucine (T244I). This nsSNP is associated with multiple sclerosis (with the ancestral allele more frequent in cases) and has been validated by meta-analysis [23]. The nsSNP that gave rise to the next most negative mean iHS was found in the collagen 11 alpha 1 (*COL11A1*) gene, associated with primary open-angle glaucoma. The ancestral allele codes for proline while the derived, protective allele codes for leucine. The third most negative was found in *SLC30A8* and is associated with Type 2 diabetes. Its ancestral allele codes for arginine whereas the derived, protective allele codes for tryptophan. We repeated this analysis in the sample with European ancestry and comparable mean iHS results were observed.

**Fig. 3:**
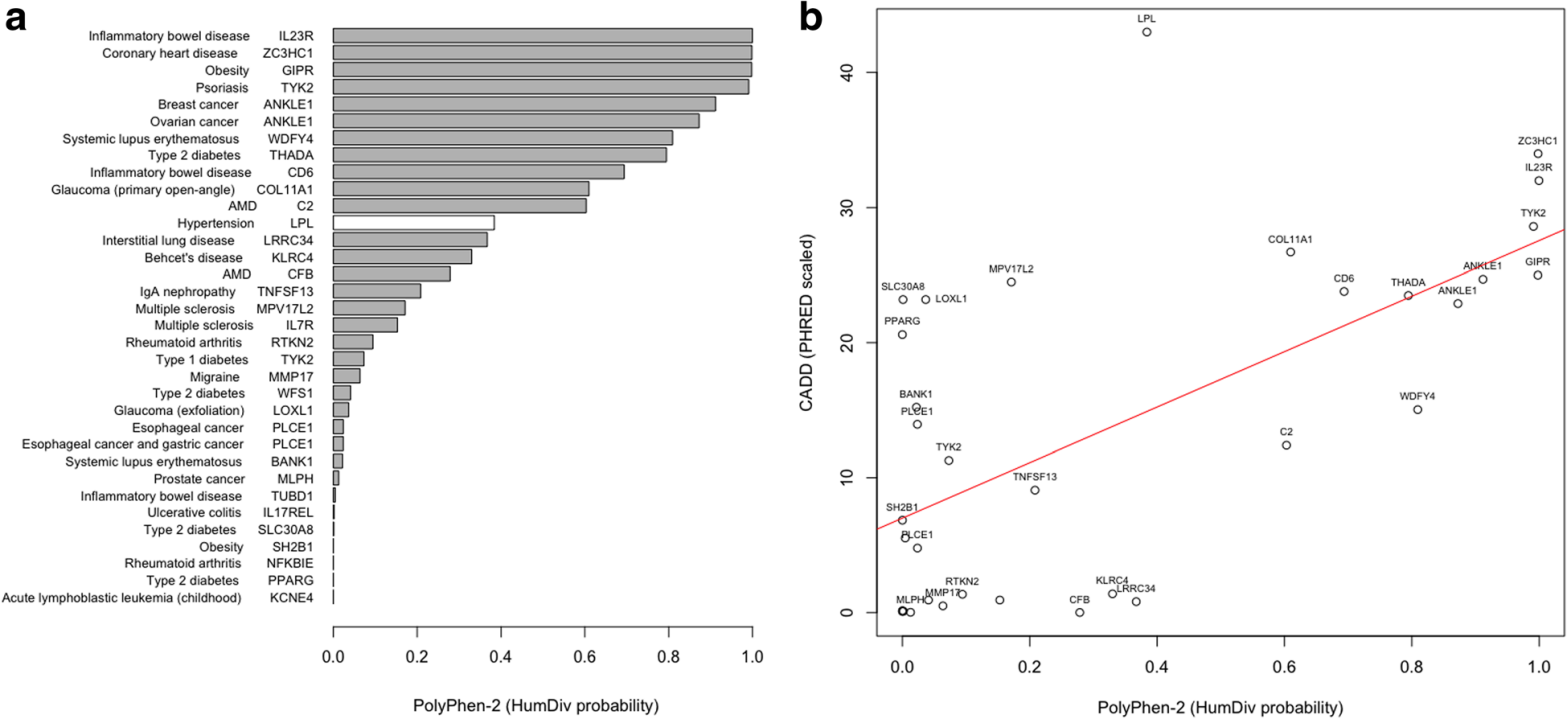
**a** Probabilities of the candidate protective variants altering their respective protein function. These probabilities are the Naïve Bayes posterior probability that the missense mutation is damaging based on the PolyPhen-2 HumDiv model. **b** Positive correlation between PolyPhen-2 and CADD scores

For each CPV, the probability of it altering protein structure was calculated using PolyPhen-2 [20]. We found that 11 of the CPVs are predicted to have a higher probability of being damaging than the *LPL* S447X variant (Fig. 3a). Note that here the word “damaging” refers to altering protein stability and function. We subsequently compared these PolyPhen-2 scores to their CADD scores [21] and find significant positive correlation (*r* = 0.63; *P* = 6.2 × 10^−5^) between the two methods (Fig. 3b and Additional file 2: Table S2).

## Discussion

Naturally occurring genetic variants with protective effect against diseases represent a valuable potential resource for translational medicine. Recent advances in gene therapy have realized this by translating the protective effect of one such variant into an effective therapeutic strategy [7]. In this work we identified a total of 34 CPVs, having the potential to protect against an array of common diseases. Two of these were identified from an analysis of AMD genetic risk factors, then 32 more were identified through the GWAS catalogue. This is not an exhaustive list because it is unlikely that a recent GWAS has the power to detect all disease-associated nsSNPs, especially rare ones. In addition we only sought those nsSNPs that were directly reported in the GWAS catalogue and thus any causal nsSNP that is captured by a proxy SNP will have been also overlooked by our present method. Linkage disequilibrium incurs a further caveat to this study; for instance, it is possible that a nsSNP at a GWAS signal results in an amino acid change that has no effect on disease risk, but it is in high LD with a non-coding causal SNP elsewhere (e.g. in a regulatory region). Recent evidence does suggest however that coding variants are more likely to be causal than regulatory variants [24]. We also have to consider that there is a chance that a derived mutation may attain a high frequency due to drift, considering that the human race has undergone population bottlenecks [25]. Accordingly we labelled the variants identified in this study as *candidate* protective variants. In this study we limited our search to nsSNPs directly reported in the GWAS catalog, in order to maximize the likelihood of them being functional. However we note that our list is not a comprehensive catalog of all candidate protective variants; it is also possible that there are functional nsSNPs not reported in the catalog but which are in high LD with non-coding signals (lead SNPs) reported therein.

To advance from being a candidate to being confirmed as truly protective, these variants will need experimental validation based on characterisation of their mechanism of action. Also these candidate protective loci may be subjected to deeper genetic studies, sequencing larger samples of cases and controls to determine if any rare nonsense mutations are associated with the same disease. In this way one can build up an understanding of how different types of functional alterations (e.g. protein-truncation) affect disease risk. However the bioinformatics approaches similar to the one we undertook here build powerful avenues leading to discovery of protective variants for common diseases. We undertook an analysis to determine if the protective function of the derived allele has given it a selective advantage compared to the ancestral allele. We found 30 of the CSVs have a mean iHS that is more negative than the *LPL* S447X SNP. As the latter has already been successfully translated into gene therapy we conclude that this finding endorses these 30 variants as strong candidates for their protective role against disease. As these variants are still only candidates for being protective, collective further evidence of selection will be informative. The recently developed Singleton Density Score (SDS) [26] is able to detect selection that has occurred more recently than iHS and therefore would be valuable in expanding our knowledge of the selection dynamics that have acted upon these CPV loci. Finally with respect to selection we did not find any of the CPVs in a set of 86 regions of selection previously identified in the human genome [27]. However even though our 34 CPVs do not reach genome-wide significance individually, we do find that taken together their iHS scores represent a significant departure towards positive selection (*P* = 2.7 × 10^−6^).

The four CPVs that have a positive mean iHS indicate that they have undergone mild negative selection. Antagonistic pleiotropy could be one explanation for this; although they have a protective function with respect to the studied disease, the derived variant may increase the risk of another disease or present a selective disadvantage. Indeed the two nsSNPs in *APOE* (see Table 1) make up a set of haplotypes, of which one is known as ε4 and is well documented to be implicated in the pathogenesis of both AMD and Alzheimer’s disease (AD). Whereas the ε4 allele decreases an individual’s AMD risk, it increases AD risk. Thus knowledge of such antagonistic pleiotropy is very important as it represents an obstacle for therapeutic translation; whereas an intervention based on such a variant may achieve a reduction in the risk of developing one disease, it may increase the risk of another. Thus before any variant can be deemed truly protective such pleiotropic factors must be investigated, including those factors that affect selection such as how prevalent are the diseases involved and what are their ages of onset. Because the majority of the CPVs identified here are for relatively late onset diseases, which are not expected to engender strong selective pressures, the derived allele may have undergone positive selection due to providing another fitness advantage in younger individuals.

The two CPVs in *CFB* and *C2*, both protective against AMD, are of particular interest in that although they are close in physical distance (≈10kb apart) they are in linkage equilibrium with one another. If their protective effects are found to be independent (no epistasis) then an individual homozygous for the derived allele at both sites is about 10 times less likely to develop AMD than an individual homozygous for the ancestral allele at both sites. We also find these SNPs to be of interest because they show evidence of recent positive selection (Fig. 1) although the disease they protect against is a late-onset disease. We speculate therefore that these may also be protective against another disease, or offer another selective advantage. This speculation is backed by the fact that these two nsSNPs are in genes with function in the complement system, a key component of innate immunity.

Also of interest with respect to imminent translational research is the CPV identified in *SLC30A8* with the derived allele predicted to be protective against Type 2 Diabetes (T2D). Recent evidence elsewhere has validated a direct functional role of this gene in T2D pathogenesis [12]. Specifically, deep sequencing of very large samples has confirmed that rare truncating, loss-of-function mutations are protective against T2D. The authors suggested the approach based on inhibiting the protein encoded by this gene as a therapeutic strategy for T2D prevention. If a protective variant is rare then it represents a particularly valuable therapeutic opportunity because a large fraction of the population is not subjected to its protective effect. Thus if translated, a rare protective variant has the potential to benefit more of the population than if the protective variant was common (assuming those already carrying the protective variant will not experience additional protection). Motivated by this, it will also be worth exploring *non-coding* rare protective alleles such as the two recently found to decrease AMD risk at the *CFH* locus (rs191281603 and rs148553336) [28].

Bringing together the research on *SLC30A8* and *LPL* we highlight the fact that two distinct mechanisms, based on loss-of-function and gain-of-function mutation respectively, can both generate a protective variant. Determining which mechanism gave rise to a protective variant will be consequential in choosing the type of translational approach. As suggested for *SLC30A8* with respect to T2D, if loss-of-function variants are observed to be protective then a therapeutic strategy aimed at inhibiting the gene product is appropriate. On the other hand, if gain-of-function variants are observed to be protective then a gene-therapy approach is likely to be the most appropriate, as employed by the *LPL* S447X-based therapy. It is also conceivable to administer the gain-of-function protein to the site of pathogenesis.

## Conclusions

This study presents the first systematic analysis of the human genome undertaken to identify CPVs. We identified 34 such variants; the diseases they confer protection against include, but are not limited to T2D, inflammatory bowel disease, multiple sclerosis and rheumatoid arthritis. We propose that these represent highly promising translational targets, potentially accelerating the pathways to novel therapeutic strategies (e.g. gene therapy). Importantly, as the CPVs are naturally occurring they may substantially improve the effectiveness and safety of such therapeutic strategies.

To investigate the evolutionary selection upon these variants we developed a new method based on the previously proposed integrated haplotype score (iHS). Applying this we found that 30 CPVs show evidence of stronger positive selection than the LPL S447X protective variant, which has already been translated into gene therapy. We also carried out a bioinformatics analysis to ascertain the type and size of the effect that the CPV has on protein function. We found that 11 of the 34 CPVs are predicted to have a higher probability of being damaging than the LPL S447X mutation. The next step towards translation will require molecular experimental approaches to test the predictions generated by our analyses. This knowledge will in turn inform the type of translational approach to take: either a gene therapy-based approach (if protection is granted by a gain of beneficial function mutation) or molecular inhibition strategies (if protection is afforded by a loss of specific function).

## Additional files

Additional file 1: Table S1. Summary of filtering stages performed on GWAS catalog. (DOCX 59 kb)

Additional file 2: Table S2. Comparison of methods (PolyPhen-2 and CADD) to predict functional effect of CPVs. (DOCX 92 kb)

## Abbreviations

AD: Alzheimer’s Disease
AMD: age-related macular degeneration
ApoE: Apolipoprotein E
CADD: Combined Annotation-Dependent Depletion
CFB: complement factor B
CFH: complement factor H
COL11A1: collagen 11 alpha 1
CPV: candidate protective variants
EPO: Enredo-Pecan-Ortheus
GWAS: genome-wide association study
iHS: integrated haplotype score
IL7R: interleukin 7 receptor
kb: kilobase
LD: linkage disequilibrium
LPL: lipoprotein lipase
nsSNP: non-synonymous single nucleotide polymorphism
SDS: Singleton Density Score
T2D: type 2 diabetes
UCSC: University of California at Santa Cruz
