# Supplementary material for: Identification of candidate protective variants for common diseases and evaluation of their protective potential
Source: BMC Genomics. 2017 Aug 3;18:575. doi: 10.1186/s12864-017-3964-3 (PMC5543444; doi:10.1186/s12864-017-3964-3)
Supplement: Supplementary file 1 — Summary of filtering stages performed on GWAS catalog. (DOCX 59 kb) [file 12864_2017_3964_MOESM1_ESM.docx]

**Additional file 1: Table S1.** Summary of filtering stages performed on GWAS catalog

|  | Description | Associations kept |
| --- | --- | --- |
| 1 | GWAS Catalog entries which include “missense” in the context column | 636 |
| 2 | After keeping only associations for which risk allele is known (*Strongest SNP-Risk Allele* ≠ ?) | 500 |
| 3 | After keeping only associations for which ancestral allele is known | 441 |
| 4 | After keeping only associations for which the risk allele matches the ancestral allele (a protective candidate) | 132 |
| 5 | After keeping only associations in which an OR is reported (removed those with a beta value i.e. those having a *95% CI* entry with either the word “increase” or “decrease”) | 55 |
| 6 | After removing ambiguous associations due to gene on –ve strand (by scrutinizing original manuscript) | 49 |
| 7 | After removing duplicates (i.e. for associations reported by more than one GWAS) | 32 |
