# Supplementary material for: Identification of candidate protective variants for common diseases and evaluation of their protective potential
Source: BMC Genomics. 2017 Aug 3;18:575. doi: 10.1186/s12864-017-3964-3 (PMC5543444; doi:10.1186/s12864-017-3964-3)
Supplement: Supplementary file 2 — Comparison of methods (PolyPhen-2 and CADD) to predict functional effect of CPVs. (DOCX 92 kb) [file 12864_2017_3964_MOESM2_ESM.docx]

**Additional file 1: Table S2.** Comparison of methods (PolyPhen-2 and CADD) to predict functional effect of CPVs

|  |  |  | **CADD** | | **PolyPhen-2** | |  |
| --- | --- | --- | --- | --- | --- | --- | --- |
| **Position** | **Gene** | **SNP ID** | **RawScore** | **PHRED** | **HumDiv Prob** | **HumVar Prob** | **Disease** |
| chr1:67705958 | IL23R | rs11209026 | 6.621 | 32 | 1.000 | 0.995 | Inflammatory bowel disease |
| chr7:129663496 | ZC3HC1 | rs11556924 | 7.480 | 34 | 0.998 | 0.939 | Coronary heart disease |
| chr19:46181392 | GIPR | rs1800437 | 4.932 | 25 | 0.998 | 0.967 | Obesity |
| chr19:10469975 | TYK2 | rs12720356 | 6.174 | 28.6 | 0.991 | 0.939 | Psoriasis |
| chr19:17392894 | ANKLE1 | rs8100241 | 4.739 | 24.7 | 0.912 | 0.792 | Breast cancer |
| chr19:17394124 | ANKLE1 | rs2363956 | 3.307 | 22.9 | 0.873 | 0.817 | Ovarian cancer |
| chr10:50025396 | WDFY4 | rs7097397 | 1.807 | 15.03 | 0.809 | 0.292 | Systemic lupus erythematosus |
| chr2:43732823 | THADA | rs7578597 | 3.892 | 23.5 | 0.795 | 0.630 | Type 2 diabetes |
| chr11:60776209 | CD6 | rs11230563 | 4.156 | 23.8 | 0.694 | 0.472 | Inflammatory bowel disease |
| chr1:103379918 | COL11A1 | rs3753841 | 5.659 | 26.7 | 0.610 | 0.336 | Glaucoma (primary open-angle) |
| chr6:31903804 | C2 | rs9332739 | 1.323 | 12.39 | 0.603 | 0.347 | AMD |
| chr8:19819724 | LPL | rs328 | 13.736 | 43 | 0.384 | 0.080 | Hypertension |
| chr3:169518455 | LRRC34 | rs6793295 | -0.262 | 0.807 | 0.367 | 0.227 | Interstitial lung disease |
| chr12:10560957 | KLRC4 | rs2617170 | -0.150 | 1.385 | 0.330 | 0.135 | Behcet's disease |
| chr6:31914180 | CFB | rs641153 | -1.929 | 0.001 | 0.279 | 0.112 | AMD |
| chr17:7462969 | TNFSF13 | rs3803800 | 0.739 | 9.081 | 0.208 | 0.125 | IgA nephropathy |
| chr19:18304700 | MPV17L2 | rs874628 | 4.625 | 24.5 | 0.171 | 0.126 | Multiple sclerosis |
| chr5:35874575 | IL7R | rs6897932 | -0.235 | 0.925 | 0.153 | 0.019 | Multiple sclerosis |
| chr10:63958112 | RTKN2 | rs3125734 | -0.156 | 1.352 | 0.094 | 0.030 | Rheumatoid arthritis |
| chr19:10475652 | TYK2 | rs2304256 | 1.112 | 11.28 | 0.073 | 0.021 | Type 1 diabetes |
| chr12:132325239 | MMP17 | rs6598163 | -0.356 | 0.493 | 0.064 | 0.046 | Migraine |
| chr4:6303022 | WFS1 | rs1801214 | -0.235 | 0.924 | 0.041 | 0.037 | Type 2 diabetes |
| chr15:74219582 | LOXL1 | rs3825942 | 3.638 | 23.2 | 0.037 | 0.018 | Glaucoma (exfoliation) |
| chr10:96066341 | PLCE1 | rs2274223 | 0.210 | 4.782 | 0.024 | 0.040 | Esophageal cancer |
| chr10:96058298 | PLCE1 | rs3765524 | 1.616 | 13.94 | 0.024 | 0.007 | Esophageal cancer and gastric cancer |
| chr4:102751076 | BANK1 | rs10516487 | 1.837 | 15.21 | 0.022 | 0.008 | Systemic lupus erythematosus |
| chr2:238443226 | MLPH | rs2292884 | -1.032 | 0.014 | 0.013 | 0.006 | Prostate cancer |
| chr17:57963537 | TUBD1 | rs1292053 | 0.283 | 5.529 | 0.005 | 0.004 | Inflammatory bowel disease |
| chr22:50435480 | IL17REL | rs5771069 | -0.652 | 0.093 | 0.002 | 0.001 | Ulcerative colitis |
| chr8:118184783 | SLC30A8 | rs13266634 | 3.640 | 23.2 | 0.001 | 0.001 | Type 2 diabetes |
| chr16:28883241 | SH2B1 | rs7498665 | 0.428 | 6.851 | 0.000 | 0.000 | Obesity |
| chr2:223917983 | KCNE4 | rs12621643 | -0.584 | 0.136 | 0.000 | 0.001 | Acute lymphoblastic leukemia (childhood) |
| chr3:12393125 | PPARG | rs1801282 | 2.658 | 20.6 | 0.000 | 0.000 | Type 2 diabetes |
| chr6:44232920 | NFKBIE | rs2233434 | -0.690 | 0.076 | 0.000 | 0.000 | Rheumatoid arthritis |
| chr5:131995964 | IL13 | rs20541 | -3.418 | 0.001 | NA | NA | Psoriasis |
